# Estimating the potential yield and ET_c_ of winter wheat across Huang-Huai-Hai Plain in the future with the modified DSSAT model

**DOI:** 10.1038/s41598-018-32980-4

**Published:** 2018-10-18

**Authors:** Xiaopei Tang, Ni Song, Zhifang Chen, Jinglei Wang, Jianqiang He

**Affiliations:** 1 Farmland Irrigation Research Institute, Chinese Academy of Agricultural Sciences, Xinxiang, 453002 China; 2 Ministry of Agriculture and Rural Affairs Key Laboratory of Crop Water Requirement and Regulation, Xinxiang, 453002 China; 3 Institute of Water Saving Agriculture in Arid Areas of China, Northwest A&F University, Yangling, Shaanxi 712100 China

## Abstract

The DSSAT model, integrated the calibrated Hargreaves ET model and dynamic crop coefficient, was run with the generated weather data by SDSM4.2 and CanESM2 to predict the potential yield and crop water requirement (ET_C_) of winter wheat in the Huang-Huai-Hai Plain in China under RCP4.5 and RCP8.5 scenarios. The results showed that the spatial distribution of potential yield in the future under RCP4.5 and RCP8.5 were similar, characterized by an increasing trend from the northwest inland to the southeast coast. The spatial distribution of ET_C_ decreased gradually from the Shandong Peninsula to the surrounding area, and the minimum ET_C_ was observed in the southern part of Huang-Huai-Hai Plain. The potential yield, ET_C,_ and effective precipitation during winter wheat growing seasons might increase in the future under RCP4.5, while irrigation water requirements (IWR) would decrease. Under RCP8.5, the effective precipitation during the wheat growing seasons decreased first and then increased. However, the potential yield, ET_C_, and IWR of winter wheat increased first and then decreased. This study can provide some scientific evidence to mitigate the negative effects of climate change on agricultural production and water use in the Huang-Huai-Hai Plain.

## Introduction

The Huang-Huai-Hai Plain is an important base for high-quality wheat production in China, as well as food security of China. However, precipitation in this region is less than 150 mm, which can not meet the water requirement of about 450 mm for winter wheat^1^. Therefore, scientific irrigation management is the key to ensure stable and high yield of grain crops as well as high water use efficiency. The accurate estimation of crop water requirement is the key to irrigation scheduling. The IPCC fifth global climate change assessment report showed that global average temperature of 2016–2035 may rise by 0.3–0.7 °C and climate warming is expected to continue throughout the 21^st^ century compared with 1986–2005^2^. Climate change will affect crop growth season^3^, planting boundaries^4^, and cropping systems^5,6^. Relative studies showed that mean seasonal evapotranspiration (ET) of winter wheat in the North China Plain under well-watered conditions gradually increased from the 1980s to 2000s^7^ and the reference ET (ET_0_) will also increase in the future under representative concentration paths^8,9^. This study aims to explore the change of winter wheat water requirement (ET_C_) under main climate scenarios in the future across Huang-Huai-Hai Plain. The results will provide a theoretical basis for the adjustment of agricultural production pattern, optimal allocation of water resources, and scientific response to climate change impact on agricultural production.

The estimation of ET_C_ in the future has been taken more attention and the main works focus on formula and crop model methods. However, both methods need global climate models (GCMs) and downscaling methods to project local weather data. Formula method uses formula to estimate ET_0_ and then crop coefficient is used to estimate ET_C_^10–12^. Due to climate change, crop growth period and physiological traits will change, and correspondingly, the value of crop coefficient will also change^7^. While mostly, the change of crop coefficient was not considered in previous studies. Though in some studies, the thermal time method was used to estimate phenology dates and crop coefficients were set at different growth stages^10,11^, the values of crop coefficient were usually set invariable at the same crop growth stage, which would increase the uncertainties in ET_C_ estimation.

Model method uses crop models to simulate growth process and directly estimates ET_C_^13–15^. Crop models can simulate the crop basic physiological processes and response to genetic characteristics, management measures, nitrogen and water stress and other factors,

and widely used to evaluate the impacts of climate change on crops growth and yields. Now the Decision Support System for Agrotechnology Transfer (DSSAT) is one of the most influential models^16^, and has been used to study the potential effects of drought on winter wheat in Huang-Huai-Hai Plain^17^, future yield changes of spring wheat on the Canadian Prairies^18^, and production of dry beans in central America^19^.

Due to the uncertainty in simulated meteorological data through downscaling models and GCMs, for example, the accuracy of the simulated temperature was high, while the accuracy of simulated precipitation and wind were relatively low^20–22^. The default Priestley-Taylor (PT) method based on temperature in the DSSAT model was usually adopted when estimating ET_C_ in the future. However, the PT method has two disadvantages. The first is the coefficient in the PT formula is constant within a certain temperature range, and second is the crop coefficient is always lacking. A number of studies had been carried out to define the coefficient in the PT formula^23–26^ and they found the coefficient varied with growth stages and regions. Therefore, an default or constant coefficient in PT method may yield a great error on ET_0_ estimation. For example, Nielsen *et al*.^27^ and Sau *et al*.^28^ reported the default PT method in the DSSAT model usually overestimated ET_C_ in the early stages of crop growth. Marek *et al*.^29^ found the simulated ET_C_ of maize was higher than the measured one, by more than 16% under full irrigation and 40% under limited irrigation. DeJonge *et al*.^30^ proposed a dynamic formula of crop coefficient based on leaf area index and applied it to simulate the yield and ET_C_ of maize with the DSSAT model under full and limited irrigation. He found the simulation accuracy of the DSSAT was greatly improved. At present, the DSSAT model was mainly used directly to estimate ET_C_ in the future, but less research was done to improve the accuracy of DSSAT.

The objectives of this study were to (1) improve the accuracy of ET_C_ simulation of winter wheat with the DSSAT v4.6 model through integrating the calibrated Hargreaves formula^31^ and a new crop coefficient estimation method^30^, and to (2) analyze the potential yield, ET_C_ and irrigation water requirements (IWR) of winter wheat in Huang-Huai-Hai Plain under representative concentration paths RCP 4.5 and RCP8.5 scenarios.

## Results

### Parameter estimation and verification of DSSAT-CERES-Wheat

**Figure 1:**
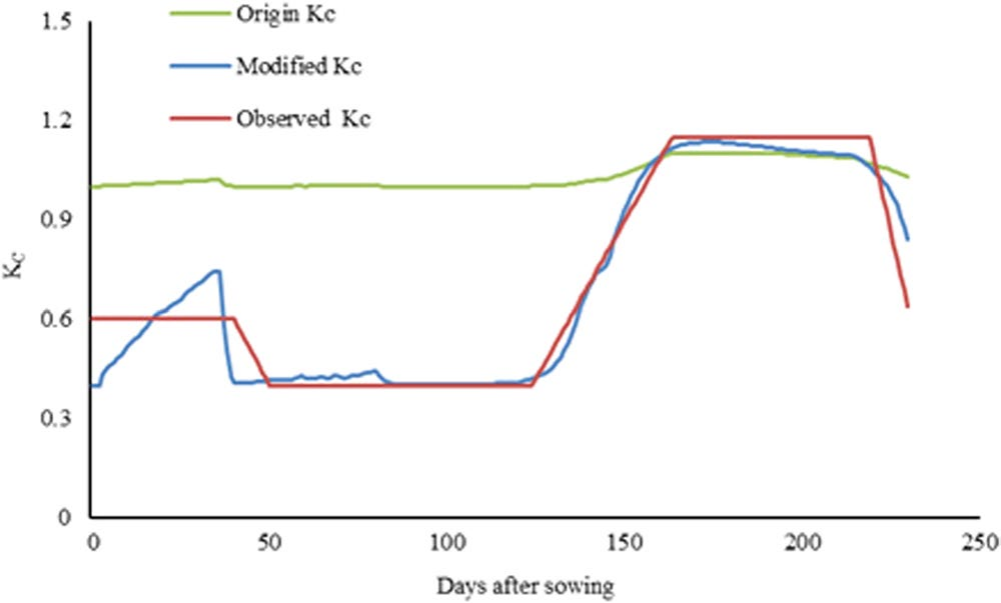
The crop coefficient curves from three methods in the 2015–2016 at the Experimental Station located in Xinxiang, Henan Province, China.

The results of genetic parameters estimation for the cultivar of ‘*Bainong 207*’ were showed in Table 1. A comparison of crop coefficient curves was showed in Fig. 1. The original K_C_ varied daily between 1.0 and 1.1, which were greater than 1.0 during the whole growth period. The modified K_C_ varied daily between 0.4 and 1.136, which were fitting well with observed ones (Fig. 1).

**Table 1:** Genetic coefficients of the winter wheat cultivar of ‘*Bainong 207*’.

Parameter	Definition	Value
P1V	Vernalization parameter/d	64
P1D	Photoperiod parameter/%	80
P5	Grain filling parameter/(°C d)	696
G1	Grain parameter at anthesis/(no g^−1^)	30.2
G2	Grain filling rate parameter/mg	45
G3	Dry weight of a single stem and spike/g	1.5
PHINT	Interval between successive leaf tip appearances/(°C d)	90

**Figure 2:**
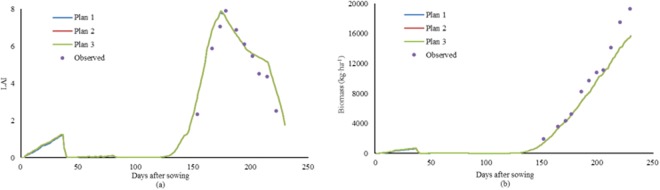
Dynamic changes of simulated LAI (**a**) and biomass (**b**) for winter wheat by the modified DSSAT models during 2015–2016 in Xinxiang, Henan Province, China. Notes: Original K_C_ is in the current version of DSSAT 4.6 for Penman-Monteith method, calculated by Eq. 17. Modified K_C_ is suggested by DeJonge *et al*.^30^, calculated by Eq. 19. Observed K_C_ came from local investigation.

The growth of winter wheat under full irrigation in 2015 and 2016 were simulated by three DSSAT modification plans (Table 2). When the genetic parameters, soil data, field management, and weather data were the same, the simulated flowering and maturity dates by the three modification plans were the same as the observed ones. However, the simulated maximum leaf area index (MLAI) by Plan 1 was the smallest, while the absolute relative error of MLAI by Plan 2 and 3 was smaller. The absolute relative error of biomass (at maturity) and yield changed very little among the three plans. The simulation results of LAI and biomass by three plans were showed in Fig. 2. Three curves of LAI looked as the same one, similar results could be found for biomass. The reason may be that only the module of evapotranspiration in DSSAT was modified, and the other modules had no change. The simulation results for both LAI and biomass were better in the middle stage of winter wheat growth and poor in the late stage of winter wheat growth. R^2^ and RMSE between the simulated LAI and observed ones by three plans were always the same, R^2^ was 0.878, and RMSE was 1.01. R^2^ between the simulated biomass and observed ones by three plans always was 0.982, while RMSE between the simulated biomass and observed ones was 1705 kg ha^−1^ by Plan 1, 1712 kg ha^−1^ by Plan 2, 1713 kg ha^−1^ by Plan 3, respectively.

**Table 2:** Results of calibration and verification of the CERES-Wheat model for winter wheat growth in the 2015–2016 growing season at the Experimental Station located in Xinxiang, Henan Province, China.

Revised item		Plan 1^a^	Plan 2	Plan 3
Anthesis date	Sim^b^	188	188	188
	Obs.	188	188	188
	ARE	0	0	0
Maturity date	Sim.	231	231	231
	Obs.	231	231	231
	ARE	0	0	0
Maximum leaf area index	Sim.	7.8	7.9	7.9
	Obs.	7.91	7.91	7.91
	ARE	1.40	0.13	0.13
Biomass (kg ha^−1^)	Sim.	15693	15653	15647
	Obs.	19306	19306	19306
	ARE	18.7	18.9	19.0
Yield (kg ha^−1^)	Sim.	10182	10256	10189
	Obs.	10168	10168	10168
	ARE	1.4	1.3	1.3

Notes: ^a^Plan 1 represents the DSSAT modification plan with the original PT model; Plan 2 the original PT model but with a dynamic Kc function; and Plan 3 the calibrated Hargreaves model and a dynamic Kc function. ^b^Sim. and Obs. represent the simulated and observed values, respectively. ARE is absolute relative error, %. And the same below.

**Figure 3:**
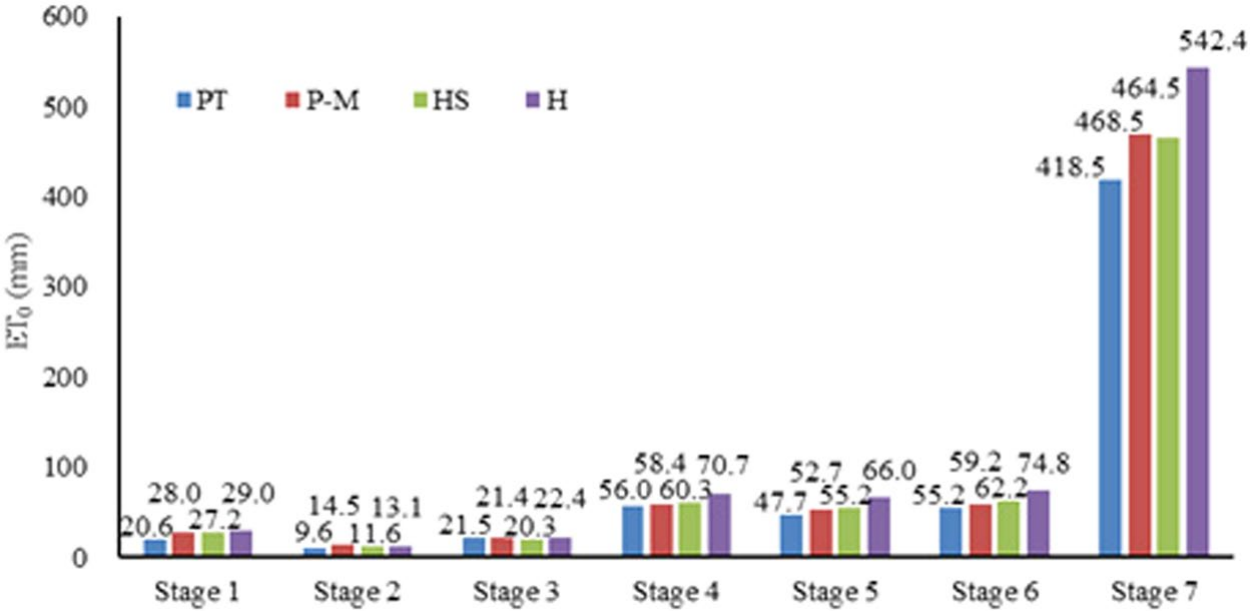
Comparison of ET_0_ estimated by different methods in the 2015–2016 different stages at the Experimental Station located in Xinxiang, Henan Province, China (mm).

Results of ET_0_ at the 7 stages in the 2015–2016 season estimated by PT, P-M, HS, H method were showed in Fig. 3. Compared with the P-M method, ET_0_ estimated by PT method were lower at all stages (except for Stage 3), ET_0_ estimated by H method were higher at all stages (except for Stage 2), ET_0_ estimated by HS method were slightly lower in Stage 1–3 (overwintering and greening) and slightly higher in Stage 4–6 (jointing, heading and grain-filling). Taking the ET_0_ estimated by P-M method as the standard, ET_0_ estimated by HS method was the most close to P-M method in Stage 1, 4, 5, 6 (overwintering, Jointing, Heading, Grain-filling), in Stage 2 (overwintering) for H method, and in stage 3 (greening) for PT method. In the whole growth periods (Stage 7), ET_0_ estimated by HS method was the most close to P-M method. Therefore, HS method had a better performance for calculating ET_0_ compared to the P-M method in Huang-Huai-Hai Plain.

**Figure 4:**
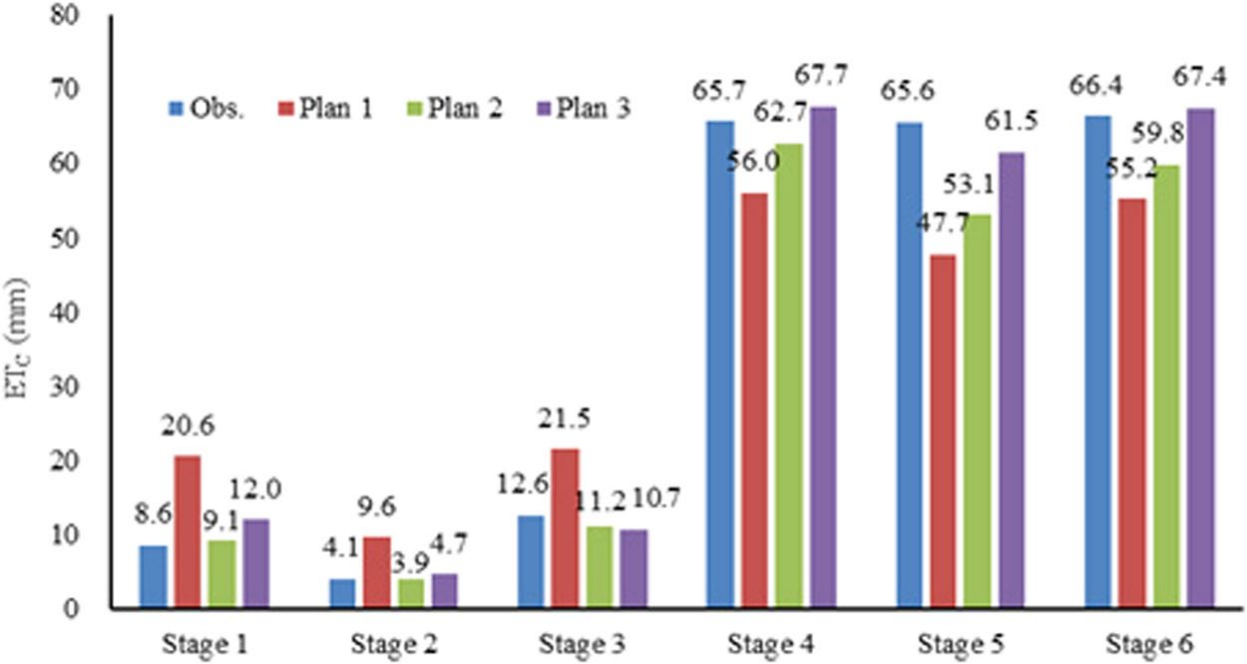
Comparison between the simulated ET_C_ and observed ET_C_ in the 2015–2016 different stages at the Experimental Station located in Xinxiang, Henan Province, China (mm).

The simulated ET_C_ and observed ET_C_ at different stages in the 2015–2016 season were showed in Fig. 4. Compared with the observed ET_C_ in different stages, the simulated ET_C_ by Plan 1 had the great difference, indicating both the default PT method and the lack of crop coefficient may cause much errors. At stages from 1 to 3, the simulated ET_C_ by Plan 1 were 12, 5.5 and 8.9 mm higher than the observed ones, respectively. However, at stages 4 to 6, the estimated ET_C_ by Plan 1 were 9.7, 17.9, 11.2 mm lower than the observed ones. The reason may be that the Plan 1 had no crop coefficient. ET_C_ in the early stages of winter wheat (stages 1–3) estimated by Plan 2 and 3 was similar and close to the observed data. However, at the late three stages (stages 4–6), ET_C_ estimated by the Plan 3 was higher than those in plan 2, and much close to the observed data with the absolute difference of −4.1 to 2.0 mm. The reason might be that the calibrated Hargreaves method mainly improve the accuracy of ET_C_ simulation at the later stages of winter wheat by integrating the crop coefficient. Considering the good performance for ET_C_ estimation in the whole growth period, this study selected Plan 3 to estimate ET_C_ in the future.

### Spatial distribution of potential yield of winter wheat under future scenarios

**Figure 5:**
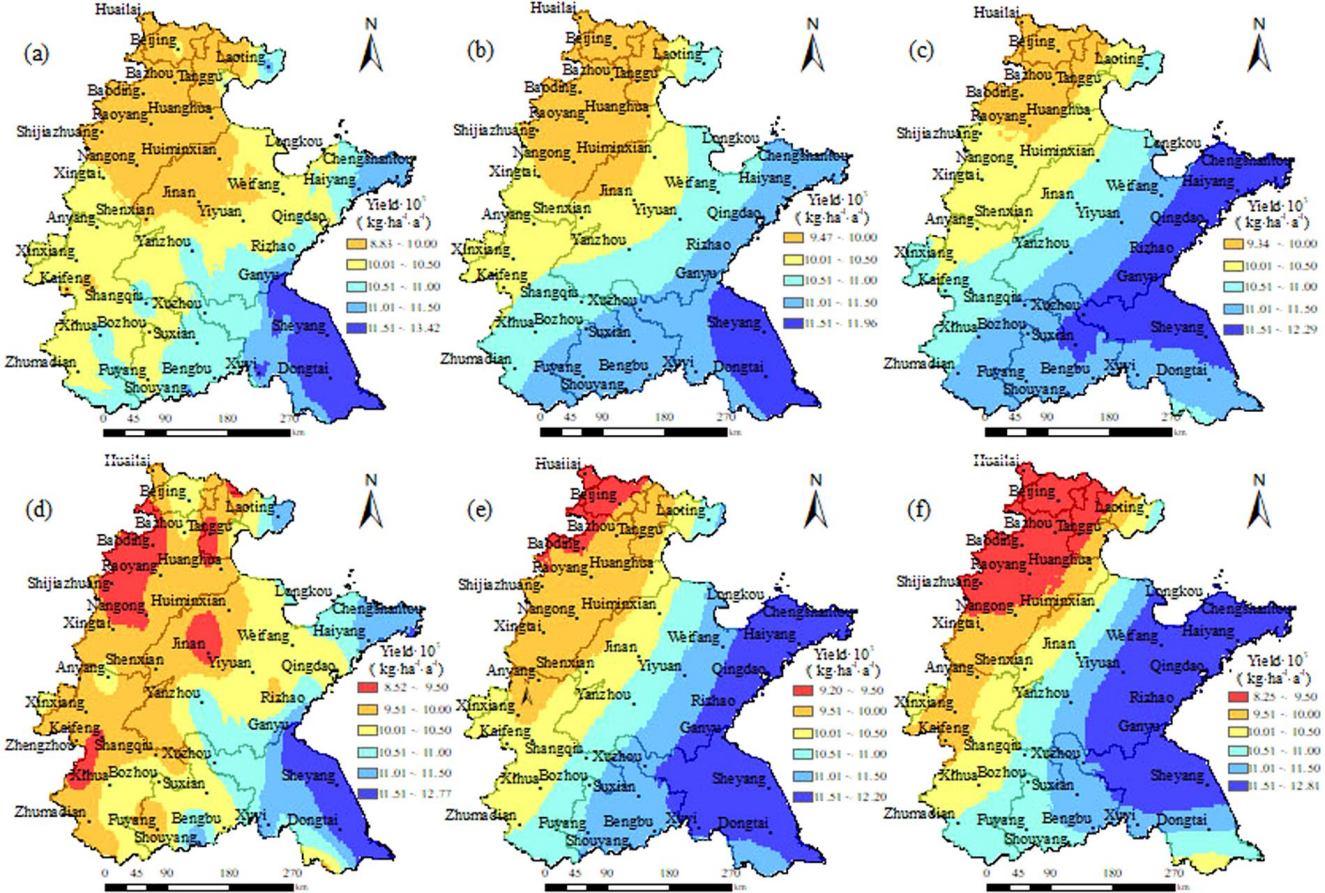
Spatial distributions of simulated winter wheat yields by the modified DSSAT model with the calibrated Hargreaves model and a dynamic Kc function in Huang-Huai-Hai Plain in 2020s, 2050s, 2080s under RCP4.5 (**a**–**c**) and RCP8.5 (**d**–**f**), respectively.

**Figure 6:**
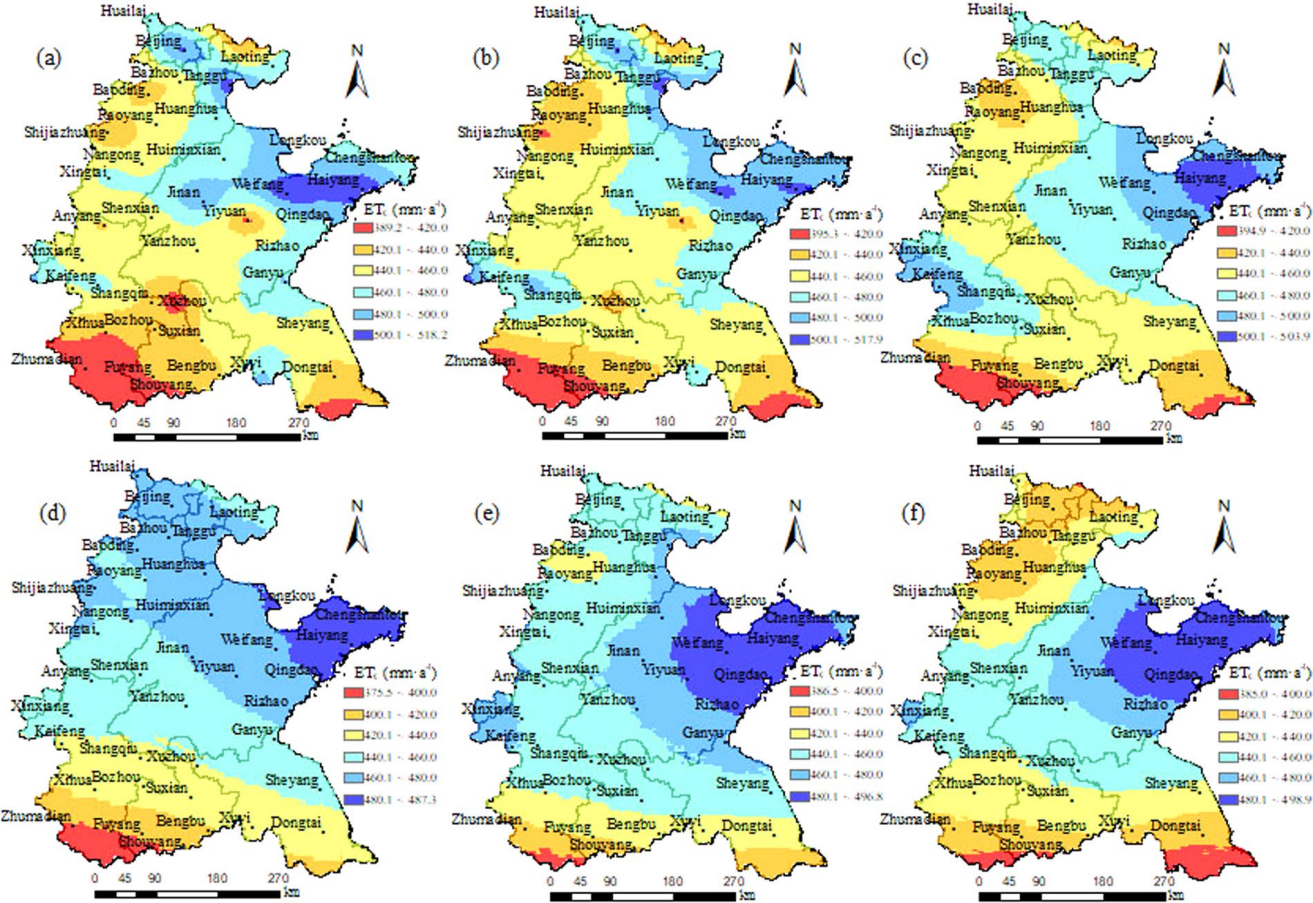
Spatial distributions of simulated ET_C_ by the modified DSSAT model with the calibrated Hargreaves model and a dynamic Kc function in Huang-Huai-Hai Plain in 2020s, 2050s, 2080s under RCP4.5 (**a**–**c**) and RCP8.5 (**d**–**f**), respectively.

The modified DSSAT model with Plan 3 was used to simulate the growth of winter wheat without water stress under main climate scenarios in the future in the Huang-Huai-Hai Plain. The spatial patterns of potential yield and ET_C_ under future scenarios are presented on Figs 5 and 6.

The spatial distributions of simulated potential yields in 2020s, 2050s, and 2080s under RCP4.5 in the Huang-Huai-Hai Plain were similar to those under RCP8.5. Yields in both scenarios increased gradually from the northwest inland to the southeast coast (Fig. 5). The area with a potential yield less than 10000 kg ha^−1^ a^−1^ under RCP4.5 was in Beijing-Tanggu region, the border between Hebei and Shandong Provinces (Fig. 5a–c). However, under RCP8.5, the area with yield less than 10000 kg ha^−1^ a^−1^ distributed in the east-central Hebei, north-central Henan, and northwestern Shandong in 2020s (Fig. 5d), then gradually decreased to the junctions of Hebei, Shandong and Henan Provinces in 2050s (Fig. 5e) and extended to Zhengzhou and Kaifeng in Henan Province in 2080s (Fig. 5f). The area with a potential yield more than 11500 kg ha^−1^ a^−1^ under RCP4.5 was near the coast of Jiangsu Province in 2020s and 2050s (Fig. 5a,b), then it expanded to the coast of Jiangsu and Shandong Provinces in 2080s (Fig. 5c). Under RCP8.5, it was near the coast of Jiangsu Province in 2020s (Fig. 5d), then gradually expanded to the inland and northern coast in 2050s (Fig. 5e) and arrived at Yiyuan in Shandong Province and Xuzhou in Jiangsu Province (Fig. 5f).

### Spatial distribution of ETC of winter wheat under future scenarios

The spatial distribution of ET_C_ in 2020s, 2050s, and 2080s under RCP4.5were also similar to those under RCP8.5 (Fig. 6), both decreased gradually from the Shandong Peninsula to the surrounding area, and the minimum ETc, generally less than 400 mm a^−1^, was observed in the southeast of Huang-Huai-Hai Plain in Jiangsu Province and the border between Henan and Anhui Provinces. Under RCP4.5, the area with the simulated ET_C_ of 440–460 mm a^−1^ accounted for about 35% of the total study area, mostly distributed in the central of Huang-Huai-Hai Plain (Fig. 6a–c). However, under RCP8.5, the area with the simulated ET_C_ of 460–480 mm a^−1^ was the largest (Fig. 6e–g). The area with the ETc more than 500 mm a^−1^ under RCP4.5 only distributed near Haiyang in Shandong Province (Fig. 6a–c), while it expanded to Weifang and Yiyuan countries, in the central part of Shandong Province under RCP8.5 (Fig. 6e–g).

### Changes of potential yield, ETC and IWR under future scenarios

The changes of main factors of winter wheat in Huang-Huai-Hai Plain in different periods were showed in Table 3. The estimated potential yield of winter wheat in the three time periods showed an increasing trend under RCP4.5, however, it increased first and then reached to the highest in 2050s, after that it decreased in 2080s under RCP8.5. The change patterns of ET_C_ were consistent with that of potential yield in the future. That’s probably because of the moderate radiation forcing and greenhouse gas concentration (2050s and 2080s under RCP4.5, 2050s under RCP8.5) were beneficial to the potential yield and ET_C_ of winter wheat, while the excessive radiation forcing and greenhouse gas concentration (2080s under RCP8.5) had an inhibitory effect. The effective precipitation during the growing period of winter wheat showed an increase trend from 195 to 208 mm a^−1^ under RCP4.5 and while it decreased first and then increased under RCP8.5. Irrigation water requirements (IWR) was different from potential yield, ETc and effective precipitation. It decreased slightly from 2020s to 2080s under RCP4.5, while it increased first and then decreased under RCP8.5.

**Table 3:** The changes of main factors of winter wheat in Huang-Huai-Hai Plain in different time in the future.

Project	RCP4.5			RCP8.5		
	2020s	2050s	2080s	2020s	2050s	2080s
potential yield (kg ha^−1^ a^−1^)	10375	10585	10676	10171	10547	10224
ET_C_ (mm a^−1^)	441.89	445.10	449.24	438.81	442.56	429.47
Effective precipitation (mm a^−1^)	195.02	200.15	208.27	194.19	184.15	191.58
IWR^a^ (mm a^−1^)	246.87	244.95	240.97	244.62	258.41	237.89

Notes: ^a^IWR represents irrigation water requirements, mm a^−1^.

## Discussions

Although the downscaling method to solve the scale mismatch problem between GCMs and crop model is well done^3,5,13–15^, the accuracy of simulated meteorological factors needs to be improved. If the meteorological data with low precision were input into the crop models, the error would accumulate for ET_C._ To study the change of ET_C_ of winter wheat in the future, the main content is to study the impacts of meteorological factors on ET_C._ Thus, accurate meteorological data are needed. So far, the method based on temperature is the most appropriate to predict ET_C_ in the future.

Climate change can affect the growth mechanism of winter wheat and ET_C_. On one hand, the increasing temperature can lead to a higher water vapor deficit and shorter growth period. The increasing concentration of CO_2_ may reduce leaf stomatal conductance of winter wheat, which consequently reduces the ET_C_^32^. On the other hand, CO_2_ fertilization and enhanced photosynthesis can increase ET_C_ of winter wheat^2,13^. Consequently, the change of ET_C_ is not remarkable^33^, which is confirmed in this study (Table 3). The greenhouse gas concentration under RCP4.5 is lower than the target level and tend to be stable, while it will increasing all the time under RCP8.5^34^. The ET_C_ of winter wheat under RCP4.5 was higher than that under RCP8.5, indicated that the higher radiation forcing and greenhouse gas concentration under RCP8.5 must inhibit the ET_C_ of winter wheat. In addition, ET_0_ in Huang-Huai-Hai Plain in 21st century under RCP4.5 and RCP8.5 showed an increasing trend^7^. The trend of ET_0_ is different from the trend of ET_C_, which may infer the complicated influence of climate change on growth period and crop coefficient of winter wheat.

In this study, the higher yield was distributed in Jiangsu and Shandong Peninsula, and the higher ET_C_ was distributed in the Shandong Peninsula in the future. However, this trend was different from that of Mo *et al*.^32^, who used the VIP model to simulate yield and ET_C_ of winter wheat in Huang-Huai-Hai region. Mo *et al*. found that relatively higher yield distributed in the Henan Province from 2000 to 2008, but ET_C_ of winter wheat in Shandong peninsula would increase in the future. With CO_2_ fertilization effects, Tao and Zhang^13^ found that the relatively higher yield distributed in southwest of Shandong and south of Henan from 1961 to 1990 and could continue to increase in the future, while the relatively higher ET_C_ distributed in southwest of Shandong and could continue to decrease in the future. The reason for these differences may exist in the GCMs, climate scenarios, downscaling methods, and crop models^13,14,18,32^.

This study hypothesized that only the winter wheat cultivar of ‘*Bainong 207*’ would be sown and the planting boundaries would not change in Huang-Huai-Hai Plain. In fact, the wheat varieties and the planting boundaries will change, which in turn influence wheat yield and ET_C_. In the past three decades, new wheat cultivars were the dominated factor for yield increase in the North China Plain^35^. Water use efficiency increased substantially from 1.0–1.2 kg m^−3^ in early 1970s to 1.4–1.5 kg m^−3^ for recent cultivars^36^. In addition, agronomic practices and technological advancement could also affect ET_C_^5^. Therefore, there must be a great uncertainty in the prediction of water requirement of winter wheat in the future.

Some researches reported that the simulation accuracy was high under full irrigation and low under limited irrigation when the DSSAT model was used to simulate crop water requirement. Marek *et al*.^37^ showed that the water requirement of maize simulated by DSSAT increased with the increase of deficit level and higher than the measured value. DeJonge^30^ showed that the simulated water requirement of maize under full irrigation was slightly lower than the measured value, while it was much higher than the measured value under limited irrigation. Then he modified the model by integrating a dynamic K_C_, and found the simulation accuracy was improved for maize water requirement under both two irrigation conditions, especially under limited irrigation.

## Conclusion

This study was to improve the DSSAT model to simulate ET_C_ of winter wheat and understand the impacts of climate change on the potential yield and ET_C_ of winter wheat in Huang-Huai-Hai Plain. The main conclusions drawn are as follows. The spatial distributions of potential yield in 2020s, 2050s, 2080s under RCP4.5 were similar to those under RCP8.5, they were yield increasing gradually from the northwest inland to the southeast coast. ET_C_ was decreasing gradually from Shandong Peninsula to the surrounding area, and the minimum ETc was predicated in the southeast of Huang-Huai-Hai Plain in Jiangsu Province and the border between Henan and Anhui Provinces. The winter wheat potential yield, ET_C_ and effective precipitation during the wheat growing period might increase from 2020s to 2080s under RCP4.5, while the IWR would decrease. Under RCP8.5, The effective precipitation during wheat season would decrease first from 2020s to 2050s, and then increase from 2050s to 2080s. The potential yield, ET_C_ and IWR would increase first and then decrease.

## Material and Methods

### Data collection

#### Weather data

Daily meteorological data of 1961–2010 for 88 weather stations in Huang-Huai-Hai Plain were collected from the China Meteorological Data Sharing Service System (http://cdc.nmic.cn/home.do) to calibrate the Hargreaves formula. The data included daily maximum and minimum temperatures, relative humidity, average wind speed and sunshine duration, and precipitation.

#### GCMs data

The Canadian Earth System Model-CanESM2 was used for future climate projection since it had been proved with a high accuracy in China^38^. The daily National Center for Environmental Prediction (NCEP) reanalysis dataset included twenty-six large-scale atmospheric variables used to calibrate and validate the SDSM model, including mean sea level pressure, near surface relative humidity, surface specific humidity, mean temperature at 2 m height. The same daily CanESM2 atmospheric variables under RCP4.5 and RCP8.5 at a resolution of 2.8125×2.8125° and for the period of 1961–2100, which were obtained from Canadian Climate Data and Scenarios (http://www.cccsn.ec.gc.ca), were used to generate future climate data.

#### DSSAT input data

Both field experiment and DSSAT model were used to evaluate the influences of climate change on the ET_C_ of winter wheat in Huang-Huai-Hai Plain. The field experiment was conducted during the winter wheat growing seasons of 2015 and 2016 at the Experimental Station located in Xinxiang (35°18′ N, 113°54′ E, 73.2 m), Henan Province, China. Climate in this region is a temperate continental climate with a mean annual temperature of 14.1 °C and an average annual rainfall of 582 mm, the groundwater level is 8 m below the ground. A main cultivated winter wheat cultivar of ‘*Bainong 207’* in northern Henan Province was sown in drills with a row spacing of 0.25m, depth of 0.05 m, and density of 400 seeds m^−2^. There was no water and fertility stress, and insect and pest were well controlled during the whole growth periods. Observed ET_C_ of each stage was calculated using a water balance method^39,40^ (Eq. 1).

1 $${{\rm{ET}}}_{{\rm{C}}}=({\rm{M}}1+{\rm{P}}+{\rm{I}}+{\rm{CR}})-({\rm{M}}2+{\rm{D}}+{\rm{R}})$$

where ET_C_ is the evapotranspiration of winter wheat (mm), M1 is the initial soil moisture (mm), P is the precipitation (mm), I is the irrigation (mm), CR is the water used by crop through capillary rise from groundwater (mm), and is negligible because the groundwater table is lower than 8 m below the ground surface^41^. M2 is the final soil moisture (mm), D is the deep drainage(mm), R is the runoff (mm).

The division of each stage was based on the phenology of winter wheat, the date of irrigation and precipitation (the value of deep drainage and runoff we couldn’t measure), and the measurement date of soil moisture. The divided stages were listed in Table 4.

**Table 4:** Division of stages for winter wheat in the 2015–2016 growing season at the Experimental Station located in Xinxiang, Henan Province, China.

Stage	Date	Total days	Phenology
Stage 1	12/18/2015-01/16/2016	30	Overwintering
Stage 2	02/06/2016-02/15/2016	10	Overwintering
Stage 3	02/24/2016-03/04/2016	10	Greening
Stage 4	03/24/2016-04/13/2016	21	Jointing
Stage 5	04/21/2016-05/05/2016	15	Heading
Stage 6	05/11/2016-05/26/2016	16	Grain-filling
Stage 7	10/18/2015-06/05/2016	230	Whole growth period

The experimental data were used for the calibration and validation of the DSSAT model, then The DSSAT model was used to simulate the growth of winter wheat from 2011 to 2100 in Huang-Huai-Hai Plain. The sowing dates of the 88 stations were obtained from local investigation and the field management was set as the same as that of the field experiment.

The weather data of field experiment, including temperature, relative humidity, average wind speed, sunshine hour, and precipitation, was collected at 0.5 hours intervals from a weather station in Xinxiang. However, for the model simulation from 2011 to 2100 the weather data were projected by SDSM4.2, including maximum temperature, minimum temperature, precipitation, and solar radiation. In addition, the solar radiation from 1961 to 2005 was calculated by the Angstrom formula as follows (Eqs 2–6).

2 $${{\rm{R}}}_{{\rm{s}}}=({{\rm{a}}}_{{\rm{s}}}+{{\rm{b}}}_{{\rm{s}}}\frac{{\rm{n}}}{{\rm{N}}}){{\rm{R}}}_{{\rm{a}}}$$

3 $${{\rm{R}}}_{{\rm{a}}}=\frac{118.08}{{\pi }}{{\rm{d}}}_{{\rm{r}}}[{{\rm{\omega }}}_{s}\,\sin ({\rm{\phi }})\sin ({\rm{\delta }})+\,\cos ({\rm{\phi }})\cos ({\rm{\delta }})\sin ({{\rm{\omega }}}_{s})]$$

4 $${{\rm{\omega }}}_{s}=\arccos [\,-\,\tan ({\rm{\phi }})\tan ({\rm{\delta }})]$$

5 $${{\rm{d}}}_{{\rm{r}}}=1+0.033\,\cos (\frac{2\pi }{{\rm{D}}}{\rm{J}})$$

6 $${\rm{\delta }}=0.409\,\sin (\frac{2\pi }{{\rm{D}}}{\rm{J}}-1.39)$$

where R_s_ is the solar short wave radiation (MJ m^−2^ d^−1^), R_a_ is the solar radiation at the top of the atmosphere (MJ m^−2^ d^−1^), n is the actual sunshine duration (h), N is the maximum possible sunshine duration (h), a_s_, and b_s_ are the regression constants, whose values change with the different meteorological conditions (e.g. humidity, dust) and solar declination (latitude, month).The suggested values of *a*_*s*_, and *b*_*s*_ in Huang-Huai-Hai Plain are 0.152 and 0.556 in spring (from March to May), 0.115 and 0.588 in summer (from June to August), 0.301 and 0.311 in autumn (from September to November), and 0.172 and 0.536 in winter (from December to the following February)^42^. And *dr* is the reciprocal of the relative distance between the sun and the earth, *ω*_*s*_ is the solar hour angle (rad), *ψ* is the geographic latitude (rad), *δ* is the solar declination (rad), *J* is Julian day of a year, D is the days of a year.

The soil data for DSSAT simulation were partially obtained from the Harmonized World Soil Database V1.2 by the Food and Agriculture Organization of the United Nations (FAO) and the International Institute for Applied Systems Analysis (IIASA). The rest of soil data were got from the 1:1000000 Chinese Soil Map by the Nanjing Institute of Soil Science, Chinese Academy of Sciences. The database contained soil physical properties of two layers of topsoil (0–30 cm) and subsoil (30–100 cm), including sand, silt and clay fractions, soil bulk density, cation exchange capacity, organic carbon content and PH. The saturated soil water content, field capacity, and permanent wilting point of each soil layer were calculated by the following formula (Eq. 7–10)^43,44^.

7 $${{\rm{\theta }}}_{{\rm{s}}}=-\,0.178+0.331{\rm{C}}+1.538{\rm{W}}-0.105{{\rm{C}}}^{2}-0.409{{\rm{W}}}^{2}-0.530{\rm{CW}}$$

8 $${{\rm{\theta }}}_{{\rm{c}}}=-\,66.58+23.24\,\mathrm{ln}({{\rm{\theta }}}_{{\rm{s}}})$$

9 $${{\rm{\theta }}}_{{\rm{w}}}=-38.75+12.53\,\mathrm{ln}({{\rm{\theta }}}_{{\rm{s}}})$$

10 $${\rm{W}}=1-\gamma /{\rm{\rho }}$$

where θ_s_, θ_c_, θ_w_ are saturated soil water content, field capacity, and wilting coefficient, respectively, C is clay fraction, W is soil porosity, γ is soil bulk density (g cm^−3^), ρ is soil specific gravity, whose suggested value is 2.65 g cm^−3^ ^45^.

### Methods

#### Statistical downscaling methods

At present, downscaling approaches include four main categories: regression methods, weather pattern approaches, stochastic weather generators, and limited-area regional climate models. Among these methods, statistical downscaling methods are the most widely used^46^. In this study, the statistical downscaling model SDSM4.2 was adopted for scale conversion. Following steps were involved to project future climatic variable. First, two sub-periods of 1961–1990 and 1991–2005 were chosen to calibrate and validate the model. Second, predictors were selected by seasonal correlation analysis, partial correlation analysis, and scatter plot analysis (α = 0.05). Third, the empirical statistical relationship between large-scale predictors and predictands (the maximum temperature, minimum temperature, solar radiation, and precipitation) was established to determine the parameters of multiple regression equation. And fourth, the coefficient of determination (R^2^) and root mean square error (RMSE) were used to quantitatively assess the performance of SDSM. Finally, the girded data for the future climate provide by CanESM2 under RCP4.5 and RCP8.5 during three future periods of the 2020s (2011–2040), the 2050s (2041–2070), and the 2080s (2071–2100) were input into the models to generate the downscaled future daily climatic series for each station.

#### Priestley-Taylor model in the DSSAT

11 $${{\rm{ET}}}_{0}={\rm{\alpha }}\times {\rm{EEQ}}$$

12 $${\rm{EEQ}}=2.04\times {10}^{-4}\cdot {{\rm{S}}}_{{\rm{R}}}-1.83\times {10}^{-4}\times {{\rm{A}}}_{{\rm{LBEDO}}}\cdot (0.6{{\rm{T}}}_{\max }+0.4{{\rm{T}}}_{\min }+29)$$

where ET_0_ is the daily reference crop evapotranspiration (mm d^−1^), EEQ is the equilibrium evapotranspiration (mm d^−1^), A_LBEDO_ is the crop albedo, T_max_ and T_min_ are the daily maximum and minimum temperature, respectively (°C), S_R_ is the daily total solar radiation (MJ m^−2^ d^−1^), α is the a coefficient of advectivity, which was set as 1.1 in the DSSAT V4.6 when temperature was between 5 and 35 °C and could be slightly smaller or larger than 1.1 (Eqs 13–14) when temperature was below 5 °C and above 35 °C.

13 $${\rm{\alpha }}=0.01\times \mathrm{EXP}(0.18\cdot ({{\rm{T}}}_{\max }+20)),\,{{\rm{T}}}_{\max } < 5$$

14 $${\rm{\alpha }}=0.05({{\rm{T}}}_{\max }-35)+1.1,\,{{\rm{T}}}_{max} > 35$$

#### Calibration of the Hargreaves model

The FAO Penman-Monteith (P-M) formula is given as follows (Eq. 15).

15 $${{\rm{ET}}}_{0}=\frac{0.408{\rm{\Delta }}\cdot ({{\rm{R}}}_{n}-{\rm{G}})+\gamma \cdot \frac{900}{{{\rm{T}}}_{{\rm{mean}}}+273}\cdot {{\rm{u}}}_{2}\cdot ({{\rm{e}}}_{{\rm{s}}}-{{\rm{e}}}_{{\rm{a}}})}{{\rm{\Delta }}+\gamma \cdot (1+0.34{{\rm{u}}}_{2})}$$

where Δ is the slope of the vapor pressure curve (kPa °C^−1^), R_n_ is the net radiation at the crop surface (MJ m^−2^ d^−1^), G is the soil heat flux density (MJ m^−2^ d^−1^), T is the air temperature at a height of 2 m (°C), U_2_ is the wind speed at a height of 2 m (m s^−1^), e_s_ is the vapor pressure of the air at saturation (kPa), e_a_ is the actual vapor pressure (kPa), γ is the psychrometric constant (kPa °C^−1^).

The Hargreaves formula is given as follows (Eq. 16).

16 $${{\rm{ET}}}_{0}=\frac{{\rm{K}}}{{\rm{\lambda }}}{({{\rm{T}}}_{{\rm{\max }}}+{{\rm{T}}}_{{\rm{\min }}})}^{{\rm{n}}}\cdot (\frac{{{\rm{T}}}_{{\rm{\max }}}+{{\rm{T}}}_{{\rm{\min }}}}{2}+{{\rm{T}}}_{{\rm{off}}})\cdot {{\rm{R}}}_{{\rm{a}}}$$

where T_max_ and T_min_ are daily maximum and minimum temperature, respectively(°C), R_a_ is the extraterrestrial radiation (MJ m^−2^ d^−1^), λ is the latency for vapouring water with suggested values 2.45 MJ kg^−1^, K, n and T_off_ are conversion coefficient, index coefficient and temperature constant with suggested values 0.0023, 0.5 and 17.8, respectively^47^.

The Hargreaves model has been widely used in various climates. Many studies have indicated that the values of the parameters of K, n and *T*_*off*_ varied from region to region. To improve the precision of the model, calibration of the three parameters of the Hargreaves model was necessary.

In this study, the nonlinear regression analysis was used to calibrate the Hargreaves formula with the software of SPSS^31^. The main process was as follows. First, the daily maximum temperature, minimum temperature, and extraterrestrial radiation were chosen as independent variable and daily ET_0_ calculated by the P-M formula were chosen as dependent variable for the 88 stations from 1961 to 2012. Next, the original values of K, n and *T*_*off*_ of Hargreaves formula were set to 0.0023, 0.5 and 17.8 in SPSS. Third, new parameter values were obtained through iterative nonlinear regression analysis. Finally, correlation index and standard error were used to quantitatively assess of accuracy of model calibration.

#### Dynamic exponential decay function of crop coefficient (K_c_)

The DSSAT4.6 mode code employs the following formula for calculation of the crop efficient K_c_ (Eq. 17).

17 $${{\rm{K}}}_{{\rm{C}}}=1.0+({\rm{EORATIO}}-1.0)\cdot \frac{{\rm{LAI}}}{6.0}$$

where K_C_ is the crop coefficient; LAI is the leaf area index; EORATIO is the ratio of increase in ET_C_ with increase in LAI, up to LAI of 6.0^28^. Despite this functionality, the accuracy of K_C_ in the current version of CERES-Wheat is still lower, for P-M method, the value of EORATIO is set to 1.1, which ensures K_C_ varies daily between 1.0 and 1.1, and for PT method, K_C_ is lacking.

DeJonge *et al*.^30^ suggested that the Kc was a dynamic exponential decay function of leaf area index (LAI) (Eq. 18).

18 $${{\rm{K}}}_{{\rm{C}}}={{\rm{K}}}_{{\rm{Cmin}}}+({{\rm{K}}}_{{\rm{Cmax}}}-{{\rm{K}}}_{{\rm{Cmin}}})\,(1-\mathrm{EXP}\,(\,-\,0.5\,{\rm{LAI}}))$$

where K_Cmin_, K_Cmax_ are the minimum and maximum K_c_ during the whole crop growth period, which were 0.4 and 1.15 for winter wheat in Huang-Huai-Hai Plain. Thus, K_c_ of winter wheat in Huang-Huai-Hai Plain was given as follows (Eq. 19).

19 $${{\rm{K}}}_{{\rm{C}}}=0.4+0.75\,(1-\mathrm{EXP}(\,-\,0.5\,{\rm{LAI}}))$$

#### The verification of main parameters and modification plan of DSSAT

In this study, the parameter estimation and verification of the DSSAT-CERES-Wheat model was carried with the DSSAT-GLUE package. The main parameters involved were P1V, P1D, P5, G1, G2, G3, and PHINT. The estimation of parameter values needed to meet three conditions. First, phenological stages or simulated dates of flowering and maturity were consistent with the actual situation. Second, the accumulation speed of simulated biomass was consistent with the actual situation through adjusting the parameters related to leaf expansion and photosynthesis. Third, the simulated final yield of winter wheat was consistent with the actual yield.

Due to the low accuracy of PT method for estimating ET_C_ of winter wheat, the calibrated Hargreaves method and a dynamic exponential decay function of K_c_ were adopted in the DSSAT4.6 model. Three DSSAT modification plans, including original PT model (Plan 1), the original PT model but with a dynamic K_c_ function (Plan 2), and the calibrated Hargreaves model and a dynamic K_c_ function (Plan 3), were used to simulate the growth of winter wheat under full irrigation in 2015 and 2016, respectively. Then a comparison between simulated ET_C_ and observed ET_C_ were made to select the modification plan with the highest accuracy. Finally, the potential yield, ET_C_ and irrigation water requirements (IWR) of winter wheat in Huang-Huai-Hai Plain under RCP 4.5 and RCP8.5 were predicted by the selected one.

#### Spatial interpolation

The ordinary Kriging method was based on the spatial position between the measured and measuring points to make a linear unbiased optimal estimation for measuring points. Then the spatial distributions of factors in the study area were analyzed by generating a spatial distribution map for the key parameters. The detailed steps of ordinary Kriging in the ArcGIS v10.2 were as follows. First, input the basic geographic information (longitude, latitude, and elevation) and factor data into ArcGIS v10.2 to get the point layer for the 88 stations. Next, combined with the surface layer of Huang-Huai-Hai Plain boundary, the ordinary Kriging interpolation method in geostatistics was used to process the data. Finally, the values of the fitting parameters were checked by semi-variance or covariance functions, and the spatial distribution map of key parameters was generated.

#### Statistical analysis

The statistics used for mode evaluation included coefficient of determination (R^2^; Eq. 20), root mean square error (RMSE; Eq. 21), and absolute relative error (ARE; Eq. 22).

20 $${{\rm{R}}}^{2}={[\frac{\sum _{{\rm{i}}=1}^{{\rm{n}}}({{\rm{Q}}}_{{\rm{i}}}-\bar{{\rm{Q}}})({{\rm{P}}}_{{\rm{i}}}-\bar{{\rm{P}}})}{\sqrt{\sum _{{\rm{i}}=1}^{{\rm{n}}}{({{\rm{Q}}}_{{\rm{i}}}-\bar{{\rm{Q}}})}^{2}\cdot \sum _{{\rm{i}}=1}^{{\rm{n}}}{({{\rm{P}}}_{{\rm{i}}}-\bar{{\rm{P}}})}^{2}}}]}^{2}$$

21 $${\rm{RMSE}}=\sqrt{\frac{1}{{\rm{n}}}\sum _{{\rm{i}}=1}^{{\rm{n}}}{({{\rm{P}}}_{{\rm{i}}}-{{\rm{Q}}}_{{\rm{i}}})}^{2}}$$

22 $${\rm{ARE}}=\frac{|{{\rm{P}}}_{{\rm{i}}}-{{\rm{Q}}}_{{\rm{i}}}|}{{{\rm{Q}}}_{{\rm{i}}}}\times 100 \% $$

where P_i_ is the i-th simulation value, Q_i_ is the i-th observation value, $\overline{{\rm{P}}}$ is the average of simulation value, $\overline{{\rm{Q}}}$ is the average of observation value; and n is the number of samples.
